# Kanavel Sign

**DOI:** 10.31662/jmaj.2024-0074

**Published:** 2024-06-03

**Authors:** Tomotaka Takanosu, Yasunori Uyama

**Affiliations:** 1 Department of Emergency and Critical Care Medicine, Jichi Medical University, Shimotsuke, Japan; 2 Internal Medicine, Fukushima Kenritsu Medical University Aizu Medical Center, Aizuwakamatsu, Japan

**Keywords:** Kanavel sign, infectious disease, pyogenic flexor tenosynovitis, emergency medicine, primary care

A 72-year-old man presented with swelling and pain in the entire left hand. Two days prior, he had visited a primary care doctor and was prescribed oral antibiotics. His left hand was swollen, and his fingers were fixed in flexion. Passive extension of the fingers elicited pain (Figure 1A and B). Magnetic resonance imaging (MRI) revealed fluid collection along the flexor tendons. Based on the Kanavel sign and MRI findings, a diagnosis of pyogenic flexor tenosynovitis (PFT) was made and irrigation debridement therapy was performed. Although mild extension impairment persisted, functional improvement was achieved to the extent. PFT progresses rapidly, necessitating early surgical intervention. Although the sensitivity of the Kanavel sign for PFT exceeds 90%, its specificity is 50% ^(1)^. Delays in surgical intervention can worsen the functional prognosis of hand movement, as observed in this case. The Kanavel sign is an important aid in early diagnosis and highlights the necessity for raising awareness of this condition in general medical practice.

**Figure 1.**
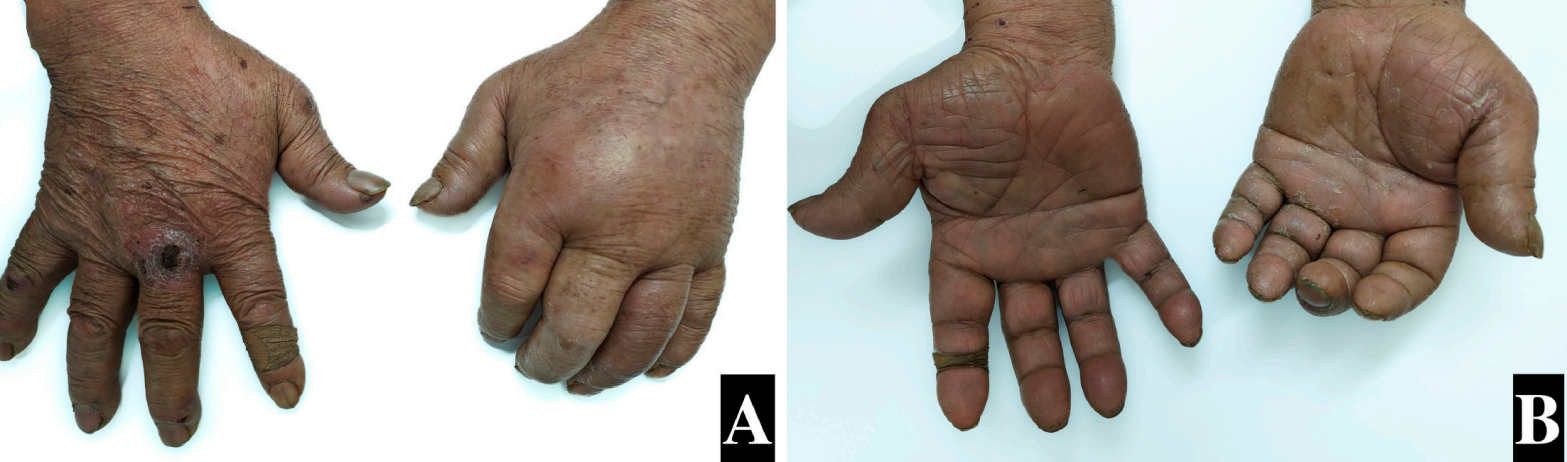
A shows patient left fingers. Fingers fixed extension position and swelling hands and fingers. Pain with finger extension, tenderness along flexor tendons, fixation in flexion, and circumferential swelling are called the Kanavel sign. B shows other views.

## Article Information

### Conflicts of Interest

None

### Author Contributions

T.T wrote manuscript. Y.U gave clinical advice.

All authors have reviewed the final draft of the manuscript.

### Approval by Institutional Review Board (IRB)

IRB approval was not required in this study.

### Informed Consent

We obtained informed consent from the patient to publish his details.
